# 

**Published:** 1849-04

**Authors:** 


					; ,
I . 1 ?-CM llwH .
Wi m
? by P. H. Austen, A. M, M. D
i* ? ? r. ?:?> ?g&S
JODRNALJ 2 ;?v
i*. ? v. --'r-,-:-
tKa Tlaltimnro r,f TWtol Snrtrorv
- ' ? "V'
by J.H.Foster,
Arti
rM-: a
-PI
-- ? i
?P' i
jbidLIOGRAPHICAL NOTICES.
A Course of Lectures ou Demal Physiology an
agwilionary df Dental Scie^ee/Biogrsqphy, Bibffograpb|r and Medi^
by Chapin A. Harris, M. D? D. D. S.,
by D. M. Reese, M. D?
?arrri8fcggTTtifia5r*5
ddingtoo, A. B , D. D. S.#
Employment of Chlorofi
Midwifery, &c.
By J. Y. Simpson,
A Manual of Auscultation and Percussion,
Translated by Francis E. Smith, M. D.,
tem
MISCELLANEOUS NOTI(
Tabular Sheet of the American Society of Dental Surg?
Dr 8. P. Hullihen's Compound Screw Forceps,
f;.*
The Pelican,
> m
rt-.W . -v ?:? * _
The Caskei and the Ribboo, or the Honors of Ether,
xon
Osseous Union of two Teeth,
Jk- ???
k--
A New Amalgam for Pilling Teeth,
MSi
Remarks on Mr. Evans' New Amalgam,
Ambler's Journal of Dental Operations,
m

				

